# Ketogenic diet for epilepsy control and enhancement in adaptive behavior

**DOI:** 10.1038/s41598-023-27373-1

**Published:** 2023-02-06

**Authors:** Omnia Fathy El-Rashidy, May Fouad Nassar, Wafaa Abdelwahab Shokair, Yasmin Gamal Abdou El Gendy

**Affiliations:** 1 Children’s Hospital, Faculty of Medicine, Ain Shams University, Cairo, Egypt; 2 Ministry of Health and Population, Cairo, Egypt

**Keywords:** Diseases, Medical research, Neurology

## Abstract

The Ketogenic Diet (KD) is gaining attention as a management line in childhood drug resistant epilepsy (DRE). The objective of this study was to highlight KD benefits for Ain Shams University (ASU) Children’s Hospital patients. This cross-sectional study included all patients at the Ketoclinic of ASU Children’s Hospital since it started. Anthropometric measurements and laboratory data were recorded. Chalfont severity score and daily frequency of epileptic attacks were used to evaluate KD efficacy. Vineland test was used to demonstrate the adaptive behavior of a selected group of patients. ASU Children’s Hospital Ketoclinic records included 143 patients. During KD therapy, the weight and height/length assessment showed significant increase with significant decrease in the severity of seizures and its frequency. There were no significant changes in the lipid profile of the patients. Vineland test showed significant improvement in the adaptive behavior in 65% of patients. The Ketoclinic data proves that KD is a tolerable, safe, and effective line of therapy for DRE in children without significant negative impact on their anthropometric measurements or lipid profile. Furthermore, the enhancement in adaptive behavior is a promising finding. It is prudent to recommend wider scale studies for longer duration to demonstrate additional cognitive benefits of KD in pediatric age group.

## Introduction

Epilepsy occurs in about 1% of the population with 60% of cases starting in the childhood period^1^**.** Epidemiological data indicate that 20–30% of patients will become refractory to therapy. Drug resistant epilepsy (DRE) is defined as seizures that cannot be controlled with adequate doses of at least two first line antiepileptic drugs, as single or combined drug therapy^2^**.**

The ketogenic diet (KD), a non-drug treatment had proven its efficacy in treatment of epilepsy in children especially in management of DRE. The KD is highly effective and reduces the incidence of seizures by 50% in half of the patients, and 90% in one third^3^***.*** In 2018 a clinical trial registration was published aiming to study various aspects of physiological health of patients on KD including cognition^4^. In the same year, Garcia-Penas^5^ reported that KD shows a positive impact on behavioral and cognitive functions. Additionally, in 2022, Koh et al.^6^, provided evidence of a neuroprotective effect of KD in the most severe DRE patients with very few remaining therapeutic options where KD can decrease the global disability after stroke.

Classic KD is also considered the treatment of choice for patient with a glucose transporter protein type 1 (GLUT1) deficiency or a pyruvate dehydrogenase (PDH) deficiency^7^. Its use in Egypt in pediatric age started early in the twenty-first century and El-Rashidy et al.^8^, was one of the earliest publications in this domain***.***

KD is a high fat, low carbohydrate, adequate protein diet that causes ketosis and leads to metabolic state that resembles the fasting state^9^***.*** It is also thought that epilepsy may represent a “metabolic disease”, and that this concept could help the development of more targeted anti-seizure drugs^10^.

It is important to exclude clinical conditions for which the KD is contraindicated (e.g., disorder of fatty acid oxidation, disorder of fatty acid transport, pyruvate carboxylase deficiency and porphyria) and assess risk factors that may complicate the use of KD (e.g., gastroesophageal reflux)^11^.

The KD has a risk of unwanted side effects. However, these issues are not usually severe, and few patients need to interrupt the diet^12^. Fluid, calorie, and protein intake are estimated based on the patient’ age, nutritional status, activity level and preference, then the type of diet and the ketogenic ratio are tailored^13^. The classical KD is deficient in some minerals and vitamins and patients on KD often use supplements with carbohydrate-free formulation^14^.

The common complications of KD are metabolic acidosis and gastrointestinal manifestations with a risk of dehydration and hypoglycemia. Less common are eating disorders, such as loss of appetite, fluid rejection, and self-induced vomiting^15^. It can also affect the immune system and cause a reduction in phagocytic index^16^.

## Aim of the work

The primary aim of the current study was to highlight the benefits of KD among patients following up in the Ketoclinic at Ain Shams University (ASU) children’s hospital in managing DRE and beyond. The secondary aim was to design a database system for registration and follow up of those patients recruited from 2013 till the end of the study and thereafter.

## Patients and methods

**Figure 1:**
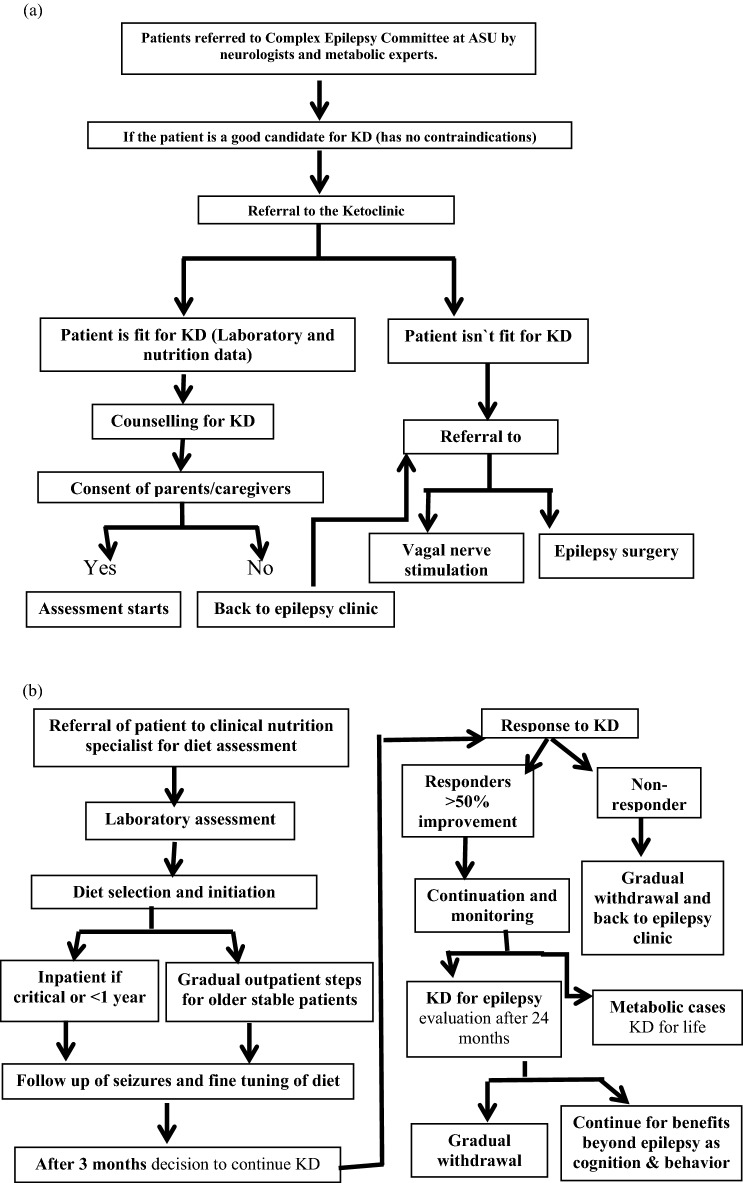
(**a**,**b**) Algorithm of the Ketoclinic protocol at the Children’s hospital Faculty of Medicine, Ain Shams University (ASU).

The current study was a descriptive cross-sectional study conducted at ASU Children’s Hospital Ketoclinic during the period from October 2017 and till March 2020. It involved all patients registered at the Ketoclinic since 2013 when it started. Patients were enrolled according to the Ketoclinic protocol (Fig. 1a,b) and those with absolute contraindications to KD were excluded from the study.

At ASU Ketoclinic the KD is initiated gradually without fasting as an outpatient unless the patient is below 1 year or in a critical condition. Ketogenic diet food recipes were used together with ketogenic formula to form a dietary plan that is tailored for each patient according to the caloric needs.

### Ethical considerations

After obtaining the approval of the ethical committee at the Pediatric Hospital, Faculty of Medicine, ASU, the aim, and steps of the study were explained to the parents and/or care givers of the patients in details and informed consents were obtained for participation while taking all privacy measures. All methods were performed in accordance with the relevant guidelines and regulations of the ethical committee. We confirm that we have read the Journal’s position on issues involved in ethical publication and affirm that this report is consistent with those guidelines.

### Methodology

A new software program designed by Compu Medical company in 1997 was implemented in the Ketoclinic to collect all data from the patients’ files and updated during patients’ visits to the Ketoclinic while only retrospective data, for children who stopped KD, was collected from archived files, and confirmed by contacting parents either face to face or by telephone. Each patient’s electronic file includes patient’s personal and socioeconomic information. It also includes family history of epilepsy, severity of the attacks (according to Chalfont seizures severity score)^17^, seizure frequency per day and the anti-epileptic drugs prescribed to each patient.

Clinical examination was done initially, periodically during the KD therapy and after its end. This included anthropometric measurements; body weight (Wt), length/height (Ht), body mass index (BMI), z-score for Wt as well as z-score for length/Ht and BMI for age plotted on WHO calculator. This data was either taken by the examiner or extracted from the files.

Two parameters were recorded for the evaluation of the efficacy of KD in the Ketoclinic patients. These were the Chalfont severity scale^17^ and the frequency of epileptic attacks per day.

Chalfont seizure severity scale assesses the severity of the convulsions by giving points on the loss of awareness during the attack, automatism, fall to ground, injury, incontinence, duration of seizure and the time to return to normal after seizure attack. By summing these points, the severity of the attack can be assessed.

Laboratory investigations included serum lipid profile [cholesterol, triglyceride, low-density lipoprotein (LDL) and high-density lipoprotein (HDL)], serum ammonia, serum lactate, extended metabolic screen, urine organic acids. Imaging procedures e.g., MRI, EEG, fundus, pelvi-abdominal ultrasound were documented as well.

The KD compliance was assessed by dietary recall during follow up visits as well as acetone in urine and random blood sugar. Compliant patients had acetone in urine ranging from + 1 to + 3 and normal random blood glucose throughout the study.

Measurement of the adaptive behavior in intellectually disabled patients by Vineland-II^18^ was performed on 26 of the Ketoclinic patients initially and after 6 months of using the KD.

### Statistical analysis

Regarding the statistical analysis IBM SPSS program (Statistical Package for Social Sciences) Version 20.0 for Windows was used to analyse the collected data which was coded before head. Frequencies and related percentages were used to present qualitative data. Means and standard deviations were used for quantitative parametric data, and median and interquartile range (IQR) were used for quantitative non-parametric data.

Continuous variables were compared by Paired-t test if parametric and Wilcoxon’s rank test if non-parametric. The verification of the statistical methods assumed a significance level of p ≤ 0.05.

## Results

The demographic data of the enrolled patients are shown in Table 1. The record of the Ketoclinic by the end of 2019 comprised 143 patients, 78 males and 75 females. According to the ketoclinic data at that time, 43 patients (30%) were still on KD, 80 patients (56%) had already stopped the diet, 15 couldn’t be reached by telephone (10.5%) and 5 (3.5%) were dead. The number of patients increased from 2013 till 2019. In 2013 and 2014 there were only 2 patients. They increased to 9 in 2015 and reached 32 in 2016, 71 in 2017, 79 in 2018 and decreased to 59 in 2019 respectively.

**Table 1:** Demographic data of the studied patients (gender, residence, mode of delivery, gestational age, consanguinity, and postnatal history).

		No	%
Gender	Female	65	45.5
	Male	78	54.5
Residence	Rural	92	64.3
	Urban	47	32.9
	unknown	4	2.8
Mode of delivery	Vaginal	41	28.7
	Cesarean section	102	71.3
Gestational age	Preterm	4	2.8
	Full term	135	94.4
	Near term	4	2.8
Consanguinity	No consanguinity	90	62.9
	Positive	53	37.1
Postnatal history	NICU admission	50	35.0
	Irrelevant	93	65.0

Regarding the diagnosis of our patients at the time of presentation, 84% were diagnosed as DRE, 30% had global developmental delay, 10.5% had cerebral palsy, 7.7% had glucose transporter 1 defect, 3.5% had post-meningitis sequel, 3.5% had post encephalitis sequel, 2.8% had neurodegenerative diseases, 2.1% had mitochondrial diseases, 2.8% had tuberous sclerosis and 2.8% were diagnosed as hypoxic ischemic encephalopathy.

Regarding the type of epileptic seizures, 113 patients had generalized epilepsy (79%), 30 had myoclonic jerks (21%), 16 had focal epilepsy (11.2%) and 7 had infantile spasm (4.9%).

In the current study 11% of patients remained on KD for ≤ 1 month, 25% of patients remained on KD for 1–3 months 18% remained on KD for 3–6 months, 13% remained on KD for 6–12 months, 20% remained on KD for 12–24 months and 13% remained on KD for > 24 months. The compliance rate was 33.6%. As regards the compliance among the patients who stopped the KD; 58% were compliant and 42% were non-compliant. Also as regards the compliance in patients who were still on the diet; 86% were compliant and 14% were non-compliant. As regards the compliance pattern of the patients during the study period, there was a noticeable increase. Causes of non-compliance in our patients are mainly diet refusal, non- availability of the diet, living in far governate with long distance and high travel costs needed to reach the ketoclinic location, school entry and old age so parents could not control their children´s diet and the final cause was the severe gastrointestinal symptoms.

The frequency of complaints and complications in our patients were recorded. Out of the studied series 34% complained of diet cost, 25.2% complained of constipation, 19.6% complained of vomiting, 12.6% complained of diet refusal, 4.9% complained of diarrhea, 4.9% of patients complained of urinary complications and 4.2% complained of difficulty in diet availability.

Regarding the age of onset of seizures in our patients, 23.1% (33 patients) had seizures since birth, 25.9% (37 patients) had seizures below 3 months of age, 19.6% (28 patients) had seizures between 3 and 6 months, 13.2% (19 patients) had seizures between 6 and 12 months and 18.2% (26 patients) had seizures beyond their first year of life.

The frequency of seizures per day showed significant decrease after 3 months of KD with p-value < 0.05 (median and IQR were 8 (5–15) and 3 (0–7) respectively) and highly significant decrease after 24 and 36 months of the diet with p-value < 0.01 (median and IQR were 0 (0–3) and 0 (0–1) respectively). On applying Chalfont seizure severity scale on our patients there was significant decrease in the severity of seizures after KD as shown in Table 2.

**Table 2:** Comparison of severity of seizure according to Chalfont score before and after KD.

Severity of seizure according to Chalfont score		No. = 143
Before KD	Mean ± SD	31.51 ± 8.19
	Range	13–67
After KD	Mean ± SD	27.14 ± 9.47
	Range	0–65
Paired t-test		7.587
p-value		0.000

On applying Vineland test to a sample of the studied patients (26 patients), 65% of them (17 patients) showed improvement in their adaptive behavior after using the KD. This result was distinct and reached statistical significance (p = 0.050) as shown in Table 3.

**Table 3:** Effect of KD on the adaptive behavior detected by using Vineland test in the studied patients.

Vineland test percentile rank		No. = 26
Before KD	Median (IQR)	11.00 (8–18)
	Range	3.00–77.00
After KD	Median (IQR)	12.50 (8–23)
	Range	4.50–95.00
Wilcoxon’s Rank test		− 1.960
p-value		0.050

**Figure 2:**
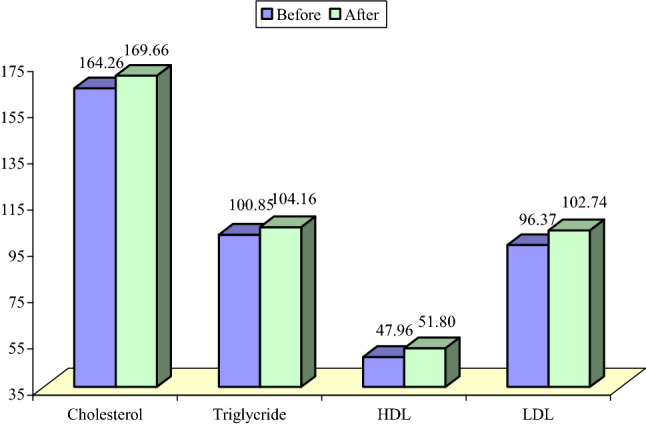
Initial and follow up values of cholesterol, high density lipoproteins (HDL), low density lipoproteins (LDL) and triglycerides in ketoclinic patients.

Concerning lipid profile of the patients (cholesterol, triglycerides, HDL and LDL) there was no significant difference in the lipid profile parameters after KD usage as shown in Fig. 2. Regarding cholesterol, two patients in the current study showed higher levels on one occasion with subsequent fall in the next measurement. Three children had elevated triglycerides sporadically (1 or 2 occasions) and only 1 patient had elevated triglycerides in several measurements during the first month of initiation of the diet.

Anthropometric measurements in the studied patients showed significant increase in the weight and z score for weight. Additionally, there were significant increases in height/length and in the z score for height/length. Finally, there were no significant increases in BMI and in z score of BMI for age as shown in Table 4.

**Table 4:** Comparison of weight, height, BMI, z score for weight, z score for height, z score for BMI before and after KD.

Anthropometric measurement		Before	After	Test value	p-value	Sig
		No. = 143	No. = 143			
Weight (kg)	Median (IQR)	13 (10–19.5)	15 (11–21)	− 6.800ǂ	0.000	HS
	Range	3–82	3.5–72			
z score for weight	Median (IQR)	− 0.51 (− 2.03 to 0.72)	− 0.98 (− 2.74 to 0.19)	− 5.296^ǂ^	0.000	HS
	Range	− 10.68 to 4.6	− 13.27 to 3.3			
Height /length (cm)	Mean ± SD	95.92 ± 23.85	101.82 ± 22.41	− 8.352^•^	0.000	HS
	Range	54–172	56–172			
z score for height	Median (IQR)	− 0.55 (− 2.3 to 0.93)	− 1.06 (− 2.49 to 0.05)	− 5.682^ǂ^	0.000	HS
	Range	− 8.52 to 11.69	− 8.53 to 3.16			
BMI [wt/(ht)^2^]	Mean ± SD	16.31 ± 4.70	16.00 ± 4.21	1.422^•^	0.158	NS
	Range	8.5–29.2	7.4–29.8			
z score for BMI	Median (IQR)	− 0.22 (− 2.85–1.43)	− 0.53 (− 2.76 to 1.14)	− 1.267^ǂ^	0.205	NS
	Range	− 16.95 to 6.01	− 24.43 to 3.92			

P-value > 0.05: Nonsignificant (NS); P-value < 0.05: Significant (S); P-value < 0.01: highly significant (HS).

^•^Paired t- test; ^‡^Wilcoxon Rank test.

## Discussion

The current study included all patients following in pediatric Ketoclinic, Children´s hospital ASU since it started in 2013. Those patients were recruited and received KD therapy according to the clinic protocol.

Regarding the frequency of complaints and complications in our patients, the major causes were gastrointestinal troubles as well as diet refusal and unavailability. This agrees with Jagadish et al.^19^ who studied 59 children with epilepsy on KD and found that adverse effects included vomiting in 24% of his patients and refusal to feed in 11%.

Regarding frequency of convulsions, there was a significant decrease after 3 months of KD and highly significant decreases after 24 and 36 months. This comes in agreement with Armeno et al.^20^ who studied 45 patients on KD and found 73% of them were responders after only three months of KD. On the other hand, Jagadish et al.^19^ initially reported a response rate of 63% at the end of the first month among his studied series but this number showed a gradual decline to 41% by the end of second year therapy.

On applying Chalfont seizure severity scale on our patients there was significant decrease in the severity of the seizure after KD. Similar finding was reported by Hallböök and collaborators^21^ who studied 18 children diagnosed as DRE on KD for 12 months, the seizure severity significantly improved at three and twelve months. The latter authors mainly attributed their findings to the decrease in duration of the convulsions.

Vineland test results of the series of studied patients showed that 65% of them had a significant increase after using the KD. Similarly, in 2001, Pulsifer and collaborators^22^ used the Child Behavior Checklist to assess behavioral and emotional problems in 65 pediatric patients with DRE after 1 year of KD therapy and reported that their mean developmental quotient showed statistically significant improvement (p < 0.05), with significant behavioral improvements in attention and social functioning. Additionally, Garcias-Penas^5^, reported that the positive neurocognitive and behavioral effect of KD in pediatric patients with epilepsy is most evident in aspects of mood, alertness and activity level, sustained attention, and reciprocal social interaction, and is not related to seizure control or a reduction in the dose or number of anti-epileptic drugs.

From another perspective, in 2016 Wu and collaborators^23^, reported that there was a positive correlation between increased cognition and the efficacy of a KD after 3 months. This is also in concordance with van Berkel et al.^24^; who conducted a systematic overview and reported that cognitive improvements are frequently reported during KD treatment in the domains of alertness, attention, and global cognition. The latter authors added that studies which used objective neuropsychological tests confirmed benefits on alertness but not in global cognition. Regarding the cause of these improvements van Berkel and associates^24^ reported that they are caused by both seizure reduction and direct effects of KD on cognition.

In the current study 11% of patients remained on KD for ≤ 1 month, 25% of patients remained on KD for 1–3 months 18% remained on KD for 3–6 months, 13% remained on KD for 6–12 months, 20% remained on KD for 12–24 months and 13% remained on KD for more than 24 months. Sharma et al.^25^ who studied 27 Indian children with epilepsy similarly reported that 37% remained on the KD after 1 year.

Concerning lipid profile of the patients there was no significant difference in any of its components after KD therapy. This agrees with a Danish study performed by Miranda et al.^26^ in which patients with refractory epilepsy received MAD showed no significant increase in free cholesterol, triglycerides, LDL and HDL cholesterol. Nevertheless, Chen^27^, who studied 47 children with intractable epilepsy found that after 3 months of KD the triglycerides and total cholesterol levels were slightly higher than the initial levels. Additionally, the HDL levels were slightly lower, and the LDL levels were significantly higher after KD therapy.

Weight assessment in our patients showed significant increase in the weight, z score for weight. On the other hand, Neal et al.^9^ found that weight z scores decreased significantly between baseline and 3, 6, and 12 months of KD therapy for their series of children with intractable epilepsy. This was attributed mainly to the caloric restriction of the KD. Weight loss was documented earlier by Sirven and collaborators^28^ who studied 11 intractable epileptic patients under treatment with KD and decrease in weight was apparent in 5 patients after 8 months on KD. Weight loss may be a result of decrease caloric intake or increased satiety effect of protein^29^***.***

Height/length assessment in the current patients showed significant increase in its value as well as the z score for age. This agrees with Vining and collaborators^30^ who reported that children grew taller during the first year on KD. In contrast, Tagliabue et al.^31^ studied 18 refractory epileptic children and they found a non-significant difference in height after 6 months KD. Additionally, Neal et al.^9^ found that height z scores showed no change at 3 months but decreased significantly by 6 and 12 months.

One suggested explanation to the improvements in the anthropometric measurements of the studied series of patients could be the fact that they were initially malnourished due to socioeconomic causes and when the KD was provided to them during the whole study period their caloric and micronutrient intakes improved which reflected well on their anthropometric measurements. Worth noting here is that although Armeno and collaborators^20^ found growth deceleration in 4 of their patients (9%) after 4 months KD therapy, the nutritional status was maintained or even improved upon follow up.

Regarding BMI, the studied series of patients showed no significant increase in BMI and in z score BMI for age and this can be explained by the proportionate increase in weight and length. This is in concordance with Perna and collaborators^32^ who found no significant changes in BMI after 12 months of KD usage in their 24 studied children.

In conclusion, registration of the Ketoclinic patients’ data using software program helped us identify the characteristics of patients on KD and highlighted the factors precluding their compliance and those ensuring their promising management outcome. ASU Children’s Hospital Ketoclinic data further proves that KD is an effective line of therapy for DRE with significant improvements in the frequency as well as the severity of convulsions without affecting their growth parameters or laboratory results. Finally, the enhancement in adaptive behavior is a promising finding and perhaps wider scale studies and longer duration of KD usage can demonstrate further cognitive benefits in treated patients.

### Study limitations

This study has its own limitations. More patients should have been subjected to the Vineland test for adaptive behavior assessment and more tests to show the cognitive benefits would have completed the picture.

## Data Availability

The authors will provide all patients’ data upon request. The corresponding author, Nassar MF, can be contacted in this regard.
